# The complete chloroplast genome sequence of *Corylopsis multiflora* Hance var*. nivea* Chang

**DOI:** 10.1080/23802359.2020.1861565

**Published:** 2021-01-27

**Authors:** Ting Lv, Shuifei Chen, Rong Zhao, Ningjie Wang, Yanming Fang

**Affiliations:** a Co-Innovation Center for Sustainable Forestry in Southern China, College of Biology and the Environment, Key Laboratory of State Forestry and Grassland Administration on Subtropical Forest Biodiversity Conservation, Nanjing Forestry University, Nanjing, China; b Research Center for Nature Conservation and Biodiversity, State Environmental Protection Scientific Observation and Research Station for Ecology and Environment of Wuyi Mountains, State Environmental Protection Key Laboratory on Biosafety, Nanjing Institute of Environmental Sciences, Ministry of Ecology and Environment, Nanjing, China

**Keywords:** *C*. *multiflora* var*. nivea*, chloroplast genome, Hamamelidaceae, Phylogeny tree

## Abstract

*Corylopsis multiflora* Hance var*. nivea* Chang is a variety of the species *C*. *multiflora* in the family Hamamelidaceae and is classed as critically endangered (CR) in the Red List of China Higher Plants. The complete chloroplast genome sequence of this taxon (as *C*. *multiflora* var. *nivea* in GeneBank, accession number: MW043717) was reported in this study. The genome size is 158,993 bp in length, consisting of a pair of inverted repeat regions (IR, 26,213bp), large single copy (LSC, 87,895bp) and small single copy (SSC, 18,672bp). A total of 133 genes were annotated that included 87 protein coding genes (PCGs), 37 transfer RNA (tRNAs), and 8 ribosome RNA (rRNAs) and 1 pseudo gene. GC content were 38.01%. The Bayesian phylogeny tree showed that *C*. *multiflora* var*. nivea* formed a monophyletic branch with *Corylopis coreana* and *Corylopsis spicata.*

*Corylopsis multiflora* Hance var*. nivea* Chang, a variety of the species *C*. *multiflora* in Hamamelidaceae, is only recorded in Fujian Province, unfortunately its habitat has been severely damaged by man disturbance and natural disasters. Therefore, this taxon has been documented as another critically endangered (CR) in the Red List of China Higher Plants (Qin et al. 2017). Although the complete chloroplast (cp) genome of a critically endangered species, *Distylium tsiangi*, in the family was recently published (Xie et al. 2020), we added a new cp genome of *C*. *multiflora* var. *nivea* to science for biodiversity conservation and phylogenetic analysis.

The fresh leaves of *C*. *multiflora* var*. nivea* were collected from a mature tree locating at Wuyi Mountains, Nanping city, Fujian Province, China (118.03°E, 27.77°N). The voucher specimen (accession number NF2017745) was reserved at the Herbarium of Nanjing Forestry University (HNFU). Total DNA was extracted using a DNeasy Plant Mini Kit (Vazyme ND607) and sequenced on the Illumina NovaSeq PE150 by Nanjing Genepioneer Biotechnologies Inc (Nanjing, China). A total of 21,534,943 clean reads were detected and assembled with SPAdes v3.10.1 (Bankevich et al. 2012). Then, the chloroplast genome was annotated using Prodigal v2.6.3 and Blastv2.6 (Zhang et al. 2019). The annotated chloroplast genome sequence of *C*. *multiflora* var. *nivea* was submitted to the NCBI GenBank (as *C. multiflora* var. *nivea*) under the accession MW043717.

The complete cp genome sequence of *C*. *multiflora* var. *nivea* contains 158,993 bp with a typical quadripartite structure, which consists of a large single-copy (LSC) region of 87,895 bp, a small single-copy (SSC) region of 18,672 bp and two inverted repeat (IR) regions of 26,213 bp. The overall G + C content of *C*. *multiflora* var. *nivea* chloroplast genome were 38.01%, while the corresponding values of the LSC, SSC, and IR regions were 36.13%, 32.55%, and 43.10%, respectively. The cp genome was composed of 133 genes, which contained 87 protein coding genes (PCGs), 37 transfer RNA genes (tRNAs), 8 ribosomal RNA genes (rRNAs) and 1 pseudo gene. Of these genes. 114 were unique and 18(*atpF, clpP, ndhA, ndhB, petB, petD, rpl16, rpl2, rpoC1, rps12, rps16, trnA-UGC, trnG-GCC, trnI-GAU, trnK-UUU, trnL-UAA, trnV-UAC, ycf3*) were duplicated in IR regions, 16 of which (ten protein coding genes and six tRNA genes) contained one intron, and two of which (*ycf3* and *clpP*) contained two introns.

To identify the phylogenetic position of *C*. *multiflora* var. *nivea* in Hamamelidaceae, we reconstructed a Bayesian phylogeny tree using MrBayes 3.2.6 (Ronquist et al. 2012). A new and highly efficient pipeline, HomBlocks, was employed to construct sequence alignment of several species from Hamamelidaceae (Bi et al. 2018). We used a Markov Chain Monte Carlo (MCMC) to run for 2,000,000 generations with four chains through two parallel searches, and each search started from a random tree (Zhang et al. 2020). Sampling was performed each 100 generations. The intial 25% of the sampled data was discarded as aging. Our results showed that *C*. *multiflora* var. *nivea* formed a monophyletic branch with *Corylopis coreana* and *Corylopsis spicata* (posterior probability =1.0; Figure 1). The cp genome sequence of *C*. *multiflora* var. *nivea* can be used to provide genomic resource for further studies.

**Figure 1.**
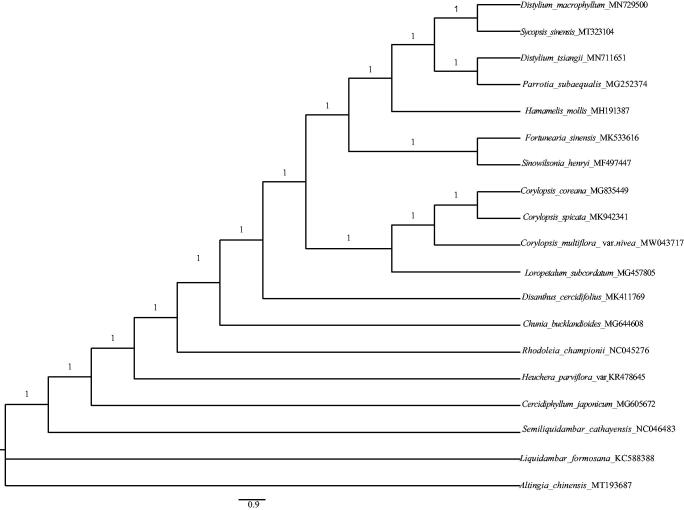
A Bayesian phylogeny tree was constructed by the cp genome sequences of 19 species from Hamamelidaceae, Saxifragaceae, Cercidiphyllaceae and Altingiaceae. The posterior probability value (1.0) is labeled for each node.

## Data Availability

The complete chloroplast genome sequence of *C*. *multiflora* var. *nivea* is deposited in the GenBank database under the accession number MW043717. The Web link is https://www.ncbi.nlm.nih.gov/
